# Impaired Recognition of Communicative Interactions from Biological Motion in Schizophrenia

**DOI:** 10.1371/journal.pone.0116793

**Published:** 2015-02-09

**Authors:** Łukasz Okruszek, Maciej Haman, Kasper Kalinowski, Monika Talarowska, Cristina Becchio, Valeria Manera

**Affiliations:** 1 Faculty of Psychology, University of Warsaw, Warsaw, Poland; 2 Department of Adult Psychiatry, Medical University of Lodz, Lodz, Poland; 3 Department of Psychology, University of Turin, Turin, Italy; 4 Department of Robotics, Brain and Cognitive Sciences, Istituto Italiano di Tecnologia, Genova, Italy; 5 CoBtek Laboratory, University of Nice—Sophia Antipolis, Nice, France; Eberhard Karls University of Tuebingen Medical School, GERMANY

## Abstract

**Background:**

Patients with schizophrenia are deficient in multiple aspects of social cognition, including biological motion perception. In the present study we investigated the ability to read social information from point-light stimuli in schizophrenia.

**Methodology/Principal Findings:**

Participants with paranoid schizophrenia and healthy controls were presented with a biological motion task depicting point-light actions of two agents either engaged in a communicative interaction, or acting independently of each other. For each stimulus, participants were asked to decide whether the two agents were communicating vs. acting independently of each other (task A), and to select the correct action description among five response alternatives (task B). Participants were also presented with a mental rotation task to assess their visuospatial abilities, and with a facial emotion recognition task tapping social cognition. Results revealed that participants with schizophrenia performed overall worse than controls both in discriminating communicative from non-communicative actions (task A) and in selecting which of the 5 response alternatives best described the observed actions (task B). Interestingly, the impaired performance of schizophrenic participants was mainly due to misclassification of non-communicative stimuli as communicative actions. Correlation analysis revealed that visuospatial abilities predicted performance in task A but not in task B, while facial emotion recognition abilities was correlated with performance in both task A and task B.

**Conclusions/Significance:**

These findings are consistent with theories of “overmentalizing” (excessive attribution of intentionality) in schizophrenia, and suggest that processing social information from biological motion does rely on social cognition abilities.

## Introduction

Schizophrenia is a major neuropsychiatric disorder which affects approximately 0.7% of the population [1]. Studies have shown that patients with schizophrenia are deficient in many aspects of social cognition, including social perception, attribution style, and theory of mind (ToM) [2]. Deficits in social cognition are now widely recognized as one of the key dimensions of impairment in schizophrenia, and have been included in MATRICS Consensus Cognitive Battery (MCCB), a standard for the assessment of cognition in schizophrenia [3]. The present study aimed to investigate the link between schizophrenia and the ability to understand social intentions carried by biological motion.

Visual processing of biological motion is a crucial aspect of social cognition, and it is of immense value for successful daily-life activities, such as social behavior and nonverbal communication [4]. A privileged way to investigate biological motion perception is the point-light methodology, consisting in portraying actions through a small number of points representing the major joints of a moving person [5]. Despite the drastic degradation of the stimulus, healthy participants are still able to recognize the portrayed actions [6, 7], to determine the identity of a figure [8], his/her gender [9, 10], his/her age [11], and his/her emotional state and personality traits [12–14]. Recently, Manera and colleagues [15] showed that observers are also able to distinguish between communicative interactions and non-communicative actions depicted through point-light displays, as well as to understand the agents’ intentions.

Disturbances in the detection of biological motion represented through point-light stimuli in patients with schizophrenia have been detected with numerous paradigms [16–20] and may be possibly linked to the aberrant activation of superior temporal sulcus (STS) [21]. For instance, schizophrenic patients have been shown to be deficient in discriminating between biological motion and scrambled motion [18] and in detecting biological masked with visual motion noise [19]. Recently, there has been a growing interest in investigating schizophrenic patients’ ability to detect *social* information from biological motion, with a special focus on emotion recognition. For instance, it has been shown that patients with schizophrenia are impaired in recognizing emotions from point-light stimuli [16], and a number of projects and consensus groups have recommended the addition of emotion recognition point-light tasks (e.g., the Perceiving Emotion Using Light Walkers Task) [14] to the standard batteries used to assess the efficacy of treatments targeting social and affective processes [22, 23]. However, to our knowledge, no study so far has investigated if schizophrenic patients are able to recognize *intentions*—for instance communicative intentions—from biological motion.

In the present study, we investigated the ability of patients with schizophrenia to recognize communicative intentions from point-light displays. Participants with paranoid schizophrenia and matched healthy controls were shown point-light video clips of two agents who were either engaged in a communicative interaction, or acting independently of each other, and asked to categorize the actions (communicative vs. non communicative), as well as to select the correct action description. As there is evidence that biological motion perception correlates with both visuo-spatial abilities (such as motor imagery) and social cognition skills [24], participants were also administered a mental rotation task and a facial emotion recognition task, which is considered as a good proxy for social cognition. Based on previous findings of impaired social cognition in schizophrenia, we hypothesized that in comparison to healthy controls, schizophrenic participants may be impaired in reading intentions from biological motion. Moreover similarly to what happens in healthy participants [24], we expected that participants’ performance in the biological motion task would possibly correlate with both visuo-spatial and emotion recognition abilities.

## Methods

### Participants

Eighteen participants (SCZ: 14M; mean age: 39y, SD: 12y; mean years of education: 12y, SD: 3y) diagnosed with paranoid schizophrenia according to ICD-10 (F20.0) criteria were recruited from Babinski Hospital in Łódź (see Table 1). Only patients with established diagnosis of schizophrenia after a minimum disease progress of two years and without any known history of neurological disorders or head injury, mental retardation, substance abuse within the past 6 months, or any comorbid psychiatric disorder participated in the study. A trained clinical psychologist (MT) rated the symptoms of each patient with the Positive and Negative Syndrome Scale [25]. All patients were receiving antipsychotic treatment at the time of the study (see Table 1).

**Table 1:** Participants’ demographic and clinical features.

	**SCZ (n = 18)** **Mean (SD)**	**HC (n = 18)** **Mean (SD)**	**SCZ versus HC** ***p***
**Age (year)**	39.1 (12.4)	35.6 (12.9)	0.32
**Male / Female**	14 / 4	14 / 4	
**Education (years)**	13.1 (3.1)	14.1 (2.6)	0.39
**IQ score**	96.8 (14.5)	109.7 (15.2)	0.01
**SPQ score**	NA	20.3 (10.1)	
**Positive and Negative Syndrome Scale**			
**Positive**	20.5 (7.6)	NA	
**Negative**	21.9 (3.9)	NA	
**Total (positive, negative, general)**	83.3 (16.4)	NA	
**CPZ equivalent (mg)**	741.4 (363.0)	NA	

Eighteen sociodemographically matched healthy persons (HC: 14M; mean age: 36y, SD 13y; mean years of education: 14y, SD: 3y) with no history of neurological or psychiatric disorders were recruited as a control group. Participants were screened for schizotypy with Schizotypal Personality Questionnaire (SPQ). The mean SPQ (M = 20.3, SD = 10.1) for the sample was much below the cut-off score indicating elevated schizotypy (41 points) [26].

All participants had normal or corrected-to-normal vision. No statistical differences were observed in age (U_(36)_ = 130.5, p = .32), education level (U_(36)_ = 189.5, p = .39), or gender (identical distribution of genders between groups). However, groups differed in IQ level (Raven Matrices: SCZ: 97+/-15 vs. HC: 110+/-15; U_(36)_ = 241.5, p<.05).

Each participant of the study provided an informed written consent. The protocol of the study was approved by University Bioethics Committee at Medical University in Łódź.

### Materials and Procedure

Each participant was examined during a single session lasting approximately 75 minutes. Upon completion of the Raven Matrices, participants completed the following tasks in a fixed order: 1) the Communicative Intention Recognition task [15], to evaluate the ability to infer communicative intentions from biological motion; 2) the PEBL version of Mental Rotation task [27], to assess visuospatial abilities, and 3) the Faces task [28] to examine the ability to recognize emotion from facial expressions.

### Communicative Intention Recognition task

**Figure 1:**
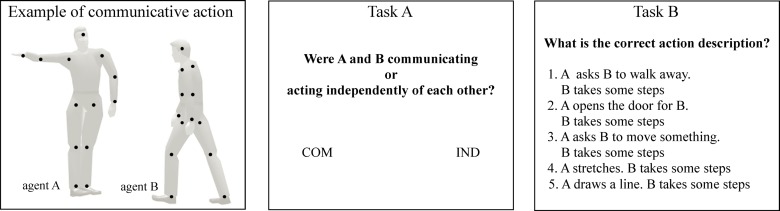
Experimental design. Example of communicative point-light action. Agent A asks B to walk away. Agent B starts walking in the required direction. Please note that n the original stimulus display, the points were white on a black background, and the silhouette depicting the human form was not visible. Task A. Participants were asked to decide whether the two agents were communicating or acting independently of each other. Task B. Participants were asked to select the correct action description among five response alternatives (correct response: 1).

Stimuli consisted of point-light actions depicting two point-light agents, each with 13 markers indicating the head, shoulders, elbows, wrists, hips, knees, and feet. Participants were presented 14 interactions in which the two agents (A and B) were engaged in a communicative interaction (e.g., agent A points out at the ceiling, agent B looks at the ceiling) and 7 control non-communicative actions, in which A and B were acting independently of each other (e.g., A drinks, B sits down). Stimuli consisted of 21 videos selected from the Communicative Interaction Database [15], and included the following actions: ‘Come closer’, ‘Get down’, ‘Go over there’, ‘Imitate me’, ‘Look at that ceiling’, ‘Look at this floor’, ‘Move over’, ‘No’, ‘Pick it up’, ‘Put it down’, ‘Sit down’, ‘Stand up’, ‘Stop’, and ‘Which one’. The non-communicative actions were created by substituting the communicative action of the first agent with a non-communicative action with the same duration (‘Turn’, ‘Jump’, ‘Sneeze’, ‘Lateral step’, ‘Drink’, ‘Stretching’, ‘Look under foot’). Stimuli were presented in randomized order. Two videos reproducing the same stimulus from 90 and 125 degrees perspectives were presented consecutively, separated by a 500ms fixation cross. After the second repetition of each video, participants were asked first asked to decide whether the two agents were communicating vs. acting independently of each other (task A), and then to select the correct action description among five response alternatives (task B; see Fig. 1). The five alternatives were assembled by replacing the correct description of agent A’s action (e.g., A asks B to walk away) with two incorrect communicative alternatives (e.g., A opens the door for B; A sks B to move something) and two incorrect non-communicative alternatives (A stretches; A draws a line; see Fig. 1, Task B).

Questions were presented on the screen until response, with no time restriction. The second question was presented only after the first question had been answered. No feedback concerning response correctness was given to the participants. The procedure was created with E-prime 2.0 software and displayed on a 17′ LCD laptop screen. The task took approximately 15–20 minutes to complete.

### Mental Rotation task

The Mental rotation task is a Psychology Experiment Building Language (PEBL) [27] version of classic Shepard’s mental rotation task [29]. Sixty four trials were presented to each participant. In each trial participants were asked to determine whether pairs of polygons displayed on screen were rotated but identical figures, or whether one polygon was the rotated mirror image of the other.

### Faces task

The Emotional Intelligence Scale—Faces task is a task validated on the Polish population which taps the ability to recognize emotion from facial expressions [28]. The task consists of 18 pictures of emotional faces including 8 positive and 10 negative emotional states (9 female faces and 9 male faces). Each emotional face expresses from one to four emotions (see [28] for more details). Each picture is associated with six emotional labels, and participants are required to indicate, for each emotion, whether it is expressed by the face or not. For instance, a picture of smiling man is presented with the following labels: “troubled” (no), “excited” (yes), “agitated” (no), “euphoric” (no), “happy” (yes), “smug” (yes). Each stimulus receives a score from 0 to 6 (1 point for every emotional label which is correctly attributed—or non-attributed—to the face), for a global score ranging from 0 to 108. No time limit was given for response selection.

## Results

### Communicative intention recognition task

**Task A: Classification of actions as communicative vs non-communicative**. Performance concerning the classification of the stimulus as communicative vs non-communicative was assessed by mean of the percentage of correct responses. Results revealed that participants with schizophrenia performed overall worse than controls in discriminating communicative from non-communicative actions (SCZ: 75%+/-9% vs. HC: 86%+/-14%; U_(36)_ = 243.0; *p*<.05). To see where participants erred, we next computed the percentage of correct responses separately for communicative and individual actions. For the communicative stimuli, no difference between groups in the percentage of correct responses was found (SCZ: 90%+/-7% vs HC: 94%+/-6%; U_(36)_ = 220.0; *p*=.07). However, SCZ participants were significantly worse than HC in the recognition of non-communicative actions (SCZ: 46%+/-32% vs HC: 69%+/-41%; U_(36)_ = 228.5; p<.05). This suggested that SCZ tended to misclassify non-communicative actions as communicative actions.

**Task B: Recognition of the specific intention**. SCZ participants performed better than chance level in selecting which of the 5 response alternatives best described the observed actions for both communicative (t(17) = 10.3, p<0.01; reference value: 20%) and non-communicative action stimuli (t(17) = 5.0, p<0.01). However, their overall performance was significantly worse than that of HC (SCZ: 57%+/-18% vs HC: 72%+/-11%; U(36) = 243.5; p<.05). Specifically, patients with SCZ performed significantly worse than HC in identifying the correct alternative for non-communicative stimuli (SCZ: 47%+/-23% vs. HC: 70%+/-15%; U(36) = 228.5; p<.05). No difference between groups was found in identifying the correct alternative for communicative stimuli (SCZ: 62%+/-17% vs HC: 73%+/-13%; U(36) = 223.5; p = .051).

To further investigate how performance in task B related to performance in task A, we re-analyze the responses in task B as communicative vs. non-communicative. Specifically, regardless of the whether participants correctly identified the correct alternative, we considered for each stimulus whether participants had selected a communicative vs. non-communicative alternative. We found that for communicative stimuli, SCZ and HC equally often selected a communicative alternative (SCZ: 88%+/-10% vs HC: 89%+/-11%; U(36) = 175.5; p = .67). In contrast, for non-communicative stimuli, the percentage of non-communicative response was significantly lower for SCZ than for HC participants (SCZ: 69%+/-21% vs HC: 83%+/-16%; U(36) = 226.0; p = .044). In line with the results of task A, this confirms the tendency of SCZ participants misclassify non-communicative stimuli as communicative.

### Mental Rotation task and Faces task

Patients with SCZ performed at the same level as HC in Mental Rotation task (SCZ: 47.0+/-12.2 vs HC: 49.3+/-11.6; U_(36)_ = 178.5; p=.52) and in Faces task score (EIS-F: SCZ: 66.1+/-11.1 vs HC: 71.8+/-11.3; U_(36)_ = 196.5; p=.28), suggesting that the two groups were comparable in terms of visuospatial and emotion recognition abilities.

### Correlations

**Figure 2:**
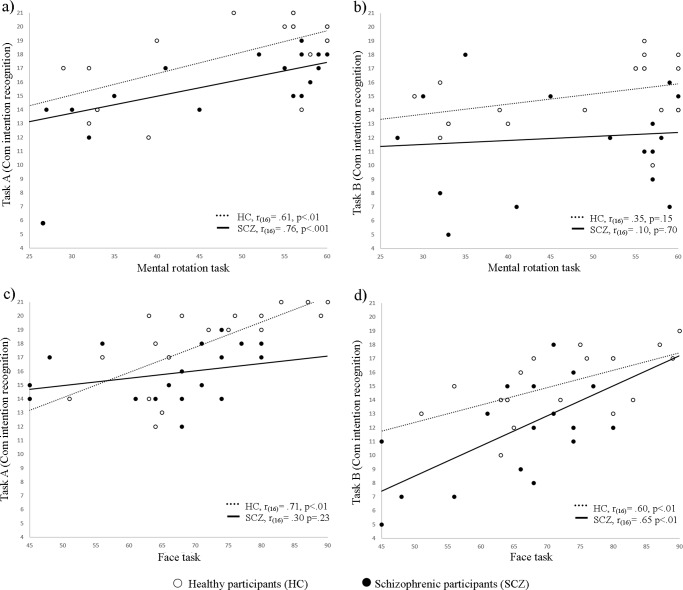
Correlations between Communicative intention recognition task (task A and task B) and Mental Rotation task (panels a and b) and Face task (panels c and d) for schizophrenic participants and healthy controls.

A significant correlations between the ability to discriminate communicative vs. non-communicative actions (task A) in the Communicative Intention Recognition task and the Mental Rotation task was observed in both the SCZ (r_(16)_ = .76; *p*<.001) and the HC group (r_(16)_ = .61; *p*<.01; see Fig. 2a). In contrast, the correlation between the ability to select the correct action alternative (task B) and the Mental Rotation task was not significant in either the SCZ (r_(16)_ = .10, *p*=.70) or the HC group (r_(16)_ = .35; *p*=.15; see Fig. 2b). A significant correlation between the Faces task score and the ability to discriminate communicative from non-communicative actions was found in HC (r_(16)_ = .71; *p*<.01), but not in SCZ (r_(16)_ = .30; *p* = .23; see Fig. 2c). The Face task score also correlated with task B in both SCZ (r_(17)_ = .65; *p*<.01) and HC (r_(16)_ = .60; *p*<.01; see Fig. 2d). No correlation was found between the IQ (as indexed by the Raven Matrices) and the other tasks (Communicative Intention Recognition task, SCZ: *p* = .35; HC: *p* = .19; Mental Rotation task, SCZ: *p* = .31; HC: *p* = .29; Face task, SCZ: *p* = .70; HC: *p* = .50). No significant correlation was found between the PANSS and the Communicative Intention Recognition Task (nor Task A nor task B), nor the Mental Rotation task, nor the Face task.

## Discussion

### Evidence for overmentalizing in schizophrenia?

In the present study we examined the ability of schizophrenic participants (SCZ) to recognize communicative and individual actions from the observation of point-light displays of two agents. Our main finding is that SCZ participants perform worse than healthy controls (HC) in discriminating between communicative and non-communicative interactions, as well as in selecting the correct response alternative describing the agents’ intentions. As revealed by inspection of errors in task A, this difference was mainly due to misclassification of non-communicative stimuli as communicative actions: in comparison to HC, SCZ patients showed a tendency to attribute communicative intentions to the agents when no communicative interaction was indeed taking place. This was further corroborated by commentaries made by the SCZ participants during the task. For instance, despite the instruction to provide a response based on what they were seeing, some participants reported that the two agents may have been communicating verbally, or through facial expressions.

A similar pattern of results was found in Task B. Specifically, SCZ participants were worse than HC in selecting the correct response alternative describing the actions of the two agents with the difference between groups reaching significance only for the non-communicative condition. The analysis of the response alternatives selected in Task B revealed that SCZ participants were more likely to choose communicative response alternatives in the non-communicative condition (i.e. to misinterpret the action of agent A as communicative) compared to HC, while no difference was observed for the communicative condition. These findings are consistent with theories of “overmentalizing” or “hyperintentionality” in paranoid schizophrenia, suggesting that SCZ patients tend to mistakenly label actions and behaviors as having more intention than they actually have [30–32]. Evidence in support of overmentalizing in schizophrenia comes from a number of behavioral studies showing that SCZ patients have a tendency to attribute intentionality also to behavior and motion patterns which are normally classified as random or mechanical by healthy participants [30; 31, 33]. Additionally, altered activity in the theory of mind (TOM) network has been reported in SCZ in comparison to healthy controls using functional MRI. For instance, Walter and colleagues [34] report that, in contrast to healthy controls, patients with paranoid schizophrenia show activations in TOM regions also when viewing stimuli depicting mere physical causality (e.g. a ball breaking a glass). A comparable pattern has been recently reported by Backasch and collegues [32], who presented participants with a set of short videos depicting two actors manipulating objects, either with or without cooperation. Activations of the ToM network (including the medial prefrontal cortex, the posterior temporal sulcus and the angular gyrus) were observed also in response to non-cooperative behaviors. Furthermore, a correlation was found between activations in the ToM network in the non-cooperative condition and the severity of the patients’ delusional symptomatology. Possibly due to to two outliers with low PANSS scores and high number of over-attribution mistakes, here we did not find a significant correlation between the PANSS (positive symptoms) and the tendency to erroneously attribute communicative intentions to the agents (task A) (r_(16)_ = .44; *p* = 0.09). Future studies, however, should remain open to the possibility that positive symptoms correlate with intention recognition deficits.

### Recognizing intentions from biological motion: correlations with visuo-spatial and emotion recognition abilities

In our study, HC and SCZ participants performed at the same level in the Mental Rotation task, suggesting that SCZ’s lower performance in the Intention Recognition task was not simply due to a general deficit in visuo-spatial skills. As revealed by the correlation analysis, however, performance in the two tasks was not independent. Specifically, classification of actions as communicative vs. non-communicative (task A), but not selection of the correct action alternative (task B), correlated with visuo-spatial abilities in both HC and SCZ participants. In line with previous studies, these results suggest that visuo-spatial abilities may contribute to extraction of visual information from biological motion [35]. However, they may not be sufficient to discriminate the specific intentions of the point-light agents, an ability that most likely requires the intervention of mentalizing and social cognition skills [36–38].

Consistently with this interpretation, we found that the ability to select the correct action description (task B) correlated with facial emotion recognition ability (as indexed by the Faces task) in both HC and SCZ participants. Facial emotion and biological movement recognition are dubbed as a two of the most studied hallmarks of social perception deficits in schizophrenia [2]. However, the relationship between these two abilities in patients with schizophrenia is still unclear. Kim [39] did not find association between the results of the Reading the Mind in Eyes task and sensitivity of detection of biological motion, while Brittain and his collaborators [16] observed a significant correlation between the ability to recognize biological motion and the results of the Half-Profile of Nonverbal Sensitivity [40]. Our findings suggest that recognition of biological motion may to some degree tap in social cognition. Further research should examine whether the ability to infer social information from biological motion correlates with other social cognition abilities, such as verbal and non-verbal Theory of Mind [41], and emotion recognition starting from body movements [42, 43].

### Limitations and future research directions

Despite our study may contribute to shed initial light on the ability of SCZ participants to detect social information from biological motion, several limitation should be addressed. First, our sample size was relatively small. Even though we tried to select a homogeneous sample of participants (e.g., we included only patients with a well-established diagnosis of paranoid schizophrenia), and we performed only non-parametric—and thus more robust—statistics, the statistical power of our analyses remains rather small. Second, we employed an explicit intention recognition task. Future studies should investigate whether similar results can be obtained employing implicit intention recognition measures [36, 37], which are less sensitive to response biases. Third, in our study the number of communicative and non-communicative stimuli was not balanced, and this may have influenced participants’ response patterns. Finally, we employed only behavioral measures. Future studies employing fMRI may help to clarify whether differences in processing communicative interactions at the behavioral level reflect differences in the neural networks underlying the processing of communicative and non-communicative actions.

## Supporting Information

S1 Table Demographic information, Communicative Intention Recognition task, Mental Rotation task and Emotional Intelligence Scale—Faces scores for each participant. (SAV)
